# Current Steering Using Multiple Independent Current Control Deep Brain Stimulation Technology Results in Distinct Neurophysiological Responses in Parkinson’s Disease Patients

**DOI:** 10.3389/fnhum.2022.896435

**Published:** 2022-06-02

**Authors:** Jana Peeters, Alexandra Boogers, Tine Van Bogaert, Robin Gransier, Jan Wouters, Bart Nuttin, Myles Mc Laughlin

**Affiliations:** ^1^Experimental Oto-rhino-laryngology, Department of Neurosciences, Leuven Brain Institute, KU Leuven, Leuven, Belgium; ^2^Department of Neurology, UZ Leuven, Leuven, Belgium; ^3^Experimental Neurosurgery and Neuroanatomy, Department of Neurosciences, Leuven Brain Institute, KU Leuven, Leuven, Belgium; ^4^Department of Neurosurgery, UZ Leuven, Leuven, Belgium

**Keywords:** movement disorders, Parkinson’s disease, deep brain stimulation, multiple independent current control, electroencephalography, evoked potentials

## Abstract

**Background:**

Deep brain stimulation (DBS) is an effective neuromodulation therapy to treat people with medication-refractory Parkinson’s disease (PD). However, the neural networks affected by DBS are not yet fully understood. Recent studies show that stimulating on different DBS-contacts using a single current source results in distinct EEG-based evoked potentials (EPs), with a peak at 3 ms (P3) associated with dorsolateral subthalamic nucleus stimulation and a peak at 10 ms associated with substantia nigra stimulation. Multiple independent current control (MICC) technology allows the center of the electric field to be moved in between two adjacent DBS-contacts, offering a potential advantage in spatial precision.

**Objective:**

Determine if MICC precision targeting results in distinct neurophysiological responses recorded *via* EEG.

**Materials and Methods:**

We recorded cortical EPs in five hemispheres (four PD patients) using EEG whilst employing MICC to move the electric field from the most dorsal DBS-contact to the most ventral in 15 incremental steps.

**Results:**

The center of the electric field location had a significant effect on both the P3 and P10 amplitude in all hemispheres where a peak was detected (P3, detected in 4 of 5 hemispheres, *p* < 0.0001; P10, detected in 5 of 5 hemispheres, *p* < 0.0001). *Post hoc* analysis indicated furthermore that MICC technology can significantly refine the resolution of steering.

**Conclusion:**

Using MICC to incrementally move the center of the electric field to locations between adjacent DBS-contacts resulted in significantly different neurophysiological responses that may allow further precision of the programming of individual patients.

## Introduction

Deep brain stimulation (DBS) is an effective therapy for medication-refractory movement disorders such as Parkinson’s disease (PD) (Limousin et al., 1998; Lyons, 2011; Kalia et al., 2013; Fasano et al., 2014). This treatment involves electrical stimulation through an electrode array (i.e., the DBS lead) implanted in a deep brain structure. For PD patients, the lead is most often implanted in the dorsolateral subthalamic nucleus (STN). Careful selection of optimal stimulation parameters is critical in ensuring an effective clinical outcome. The parameter space is large and includes stimulation intensity, stimulation rate, pulse width, configuration, and polarity (Wagle Shukla et al., 2017; Santaniello et al., 2018; Koeglsperger et al., 2019). The advent of directional leads and multiple independent current controlled (MICC) DBS now allow for even more precise targeting of the electric field toward the target region and away from side effect-causing regions (Steigerwald et al., 2019). These advances have been shown to improve clinical outcomes (Pollo et al., 2014; Steigerwald et al., 2016; Dembek et al., 2017; Krack et al., 2019; Vitek et al., 2020), but due to variance in lead placement, parameter space and patient heterogeneity, programming individual patients to determine the optimal electric field location has become increasingly time-consuming and labor-intensive (Sasaki et al., 2021). To improve this, better understanding of the different neural circuits activated with the different DBS parameters could help elucidate how DBS affects specific neural networks, and thereby it could guide DBS programming. DBS activation has been investigated in PD patients through evoked potential (EP) recordings using electroencephalography (EEG) (Walker et al., 2012) and electrocorticography (Miocinovic et al., 2018). These studies suggest that an EP recorded around 3 ms post stimulus (P3) may be important for predicting clinical outcomes.

Furthermore, in a recent study performed by our research group (Peeters et al., 2021), we recorded, in addition to a P3 peak, a peak around 10 ms post stimulus (P10) using EEG in eight patients implanted with directional leads. In that study, we showed that changing the stimulation contact using a single current source approach significantly affected the amplitude of both P3 and P10. Furthermore, combining the EEG with fused pre-operative MR and postoperative CT images showed that P3 was largest when stimulating on the dorsal DBS-contacts closest to dorsolateral STN and P10 the largest when stimulating on the ventral DBS-contacts closest to the substantia nigra pars reticulata (SNr). This suggests that P3 could serve as a biomarker for contacts closest to the dorsolateral STN, while P10 may be useful for predicting which contacts will give SNr-related side effects. Thus, EEG-based EPs could provide useful information to objectively guide programming in patients implanted with directional leads.

Multiple independent current control (MICC) technology now provides the ability to divide the total current delivered independently over two or more DBS-contacts. In the present study, we investigated if using MICC to move the electric field vertically in small incremental steps would result in distinct changes in the EEG recorded P3 and P10 amplitudes. If successful, P3 and P10 amplitudes could serve as a biomarker to evaluate the precise targeting of electric field locations for optimal clinical outcome. Here, we measured EEG-based EPs during low frequency (10 Hz) DBS and used MICC DBS to stimulate at sixteen different depths along tightly spaced (distance of 0.5 mm between two depths) directional leads in PD patients.

## Materials and Methods

### Participants

The study was approved by the Ethics committee Research UZ/KU Leuven (S62373) and registered on Clinicaltrials.gov (NCT04658641). All participants received oral and written information and provided oral and written consent. The study was conducted in conformity with the Declaration of Helsinki, the Belgian law of May 7th 2004 on experiments on the human person and in agreement with Good Clinical Practice guidelines.

Participants that met the “UK PD Society Brain Bank Clinical Diagnostic Criteria” for the diagnosis of idiopathic PD, were included in the study (Postuma et al., 2015). Directional leads (Vercise Cartesia^®^, Boston Scientific; BSC, Valencia, CA, United States) were bilaterally implanted in the STN and subcutaneously connected to the implantable pulse generator (IPG; Vercise DBS Systems, BSC, Valencia, CA, United States) that has MICC technology designed to allow for refined division of the total current over multiple DBS-contacts (Boston Scientific Corporation, 2018). The DBS-leads consist of eight DBS-contacts with a length of 1.5 mm, separated from one another by interspaces of 0.5 mm and arranged in a tip-3-3-1 configuration (Paff et al., 2020) (distal-to-proximal axis of the electrode contact numbering of left lead: C1-C8; and the right lead: C9-C16, where “C” stands for “Contact”). The surgical procedure was performed as standard-of-care at our center using the microrecording technique (Gross et al., 2006).

Patients that already participated in the previous study (Peeters et al., 2021) were now enrolled in a follow-up study, where we tested the MICC technology (see further). Four patients participated, one of which participated twice, yielding data from both hemispheres in this patient. In total, five hemispheres were tested. All participants were asked to refrain from taking their medication 12 h prior to the study visit. Demographic data and stimulation parameters used during the experiment are summarized in Supplementary Table 1.

### Deep Brain Stimulation

First, stimulation was turned off in both hemispheres. One hemisphere was tested at a time, with the other hemisphere remaining off. Thereafter, the stimulation intensity was defined on the clinical contact (monopolar cathodic pulse with return on the case; 60 μs and 130 Hz) as the highest stimulation intensity without non-transient stimulation-induced side effects. For the experimental setup, stimulation was then decreased to 10 Hz. An *in vitro* phantom head experiment was performed as a negative control where no EPs were expected. The set-up used a head-sized watermelon, where a directional lead (Vercise Cartesia^®^, BSC, Valencia, CA, United States) was positioned approximately 6.0 cm from the surface. EEG channels were then positioned on the surface and an anterior-posterior direction was appointed depending on the location of the EEG channels. All processing steps and analyses performed on real patient datasets were repeated for the phantom head dataset.

At the start of the experiment, the electric field (which had an approximately constant volume throughout the experiment as the same stimulation intensity was applied throughout the experiment) was set at the center of the most dorsal DBS-contact (i.e., 100% on C8 for the left hemisphere and C16 for the right hemisphere) for 50 s, yielding a total of 500 epochs of 100-ms duration. Then, the electric field was moved in a ventral direction in fifteen equal steps until the most ventral DBS-contact was tested. Thus, we tested sixteen incremental positions in total. The two most distant electric field locations had a distance of 6.0 mm in total, thus equating each proportional shift in the electric field was about 0.4 mm (6.0 mm/15 steps) per step. The segmented contacts were only tested in ring mode to avoid confounding the results with horizontal steering as a variable.

### Electroencephalography and Artifact-Reduction Method

EEG recordings were performed with a 64-channel ActiveTwo BioSemi system with a sample rate of 16,384 Hz and a built-in low-pass filter with a cut-off frequency of 3,200 Hz. This EEG system uses active recording channels positioned according to the internationally standardized 10–20 system (Jasper, 1958) and referenced to the vertex EEG channel (Cz). One additional EEG channel (EXG1) was positioned on the skin on top of the implanted IPG to record the stimulation pulse, which served as a trigger channel to align EPs. Two additional EEG channels were positioned on the left (EXG2) and right (EXG3) mastoid to record the stimulation pulse at a cranial location with negligible neural responses. We stimulated each of the 16 depths for 50 s at 10 Hz, yielding a total of 500 epochs with a duration of 100 ms for each depth. Each epoch was baseline corrected by subtracting the average of a 1-ms period prior to stimulus onset. Then the epochs were averaged to get the averaged EP. We applied a combination of linear interpolation and template-subtraction to reduce the total stimulation-induced artifact. Template subtraction was based on the artifact recorded with EEG electrodes EXG2 and EXG3. Two bandpass 2nd-order Butterworth filters were applied to these EPs. One was designed for evaluation of short-latency responses with a high-pass cutoff frequency of 150 Hz and low-pass cutoff frequency of 1,000 Hz. The other was designed for evaluation of long-latency responses with a high-pass cutoff frequency of 1 Hz and low-pass cutoff frequency of 150 Hz. A more detailed description of the EEG protocol and artifact-reduction method can be found in Peeters et al. (2021).

### Software and Statistical Analysis

All data processing and statistical analyses were done in MATLAB 2021a (Mathworks, Natick, MA, United States). A significance level of 5% was used in all tests. Based on the previous study (Peeters et al., 2021), we recorded a short-latency peak at 3 ms (P3) *via* the motor cortex EEG channel ipsilateral to stimulation (i.e., F3 for left hemisphere, F4 for right hemisphere) as in this EEG channel the strongest P3 was recorded. For the same reason, we recorded a long-latency peak at 10 ms (P10) *via* the prefrontal cortex EEG channel ipsilateral to stimulation (i.e., AF7 for left hemisphere, AF8 for right hemisphere). By central limit theorem, the individual EPs recorded conform to Gaussian assumptions so parametric statistics were used (Central Limit Theorem, 2008). Thus, we used one-way ANOVA to evaluate if the MICC depth of stimulation affected the P3 and P10 peak amplitude as measured in each individual hemisphere. Each EP consisted of more than 400 epochs, thus enough data were available to perform robust statistics at the individual hemispheric level. In the previous study, a one-way ANOVA was used to investigate if increasing stimulation intensity significantly affected P3 and P10 amplitude. If no significant effect of intensity was found on the peak amplitude, no further analysis was performed in this hemisphere (see Supplementary Table 1). For the remaining hemispheres, we used one-way ANOVA to evaluate if MICC technology significantly affected EP peak amplitude. Next, to test the separability of MICC on the P3 and P10 peak amplitude between different electric field pairs (varying from one step between two immediately adjacent electric field pairs to fifteen steps between two electric field pairs). For this, a *post hoc* analysis with a Bonferroni correction for multiple comparisons was applied (MATLAB, multcompare).

To investigate the relationship between the distance from each electric field center to relevant anatomical regions, we grouped all tested hemispheres (analysis on the individual hemisphere level can be found in Supplementary Figure 5). The open-source Lead-DBS image processing pipeline (version 2.5.3, Berlin, Germany) (Horn and Kühn, 2015; Horn et al., 2019) was used for postoperative lead reconstruction analyses, allowing the determination of the specific lead position and orientation on an individual hemispheric level. We then calculated the distance between the center of each electric field and the closest voxel of certain brain regions using the Distal atlas (Ewert et al., 2018).

## Results

### Short-and Long-Latency Responses Using Multiple Independent Current Control Technology

**FIGURE 1:**
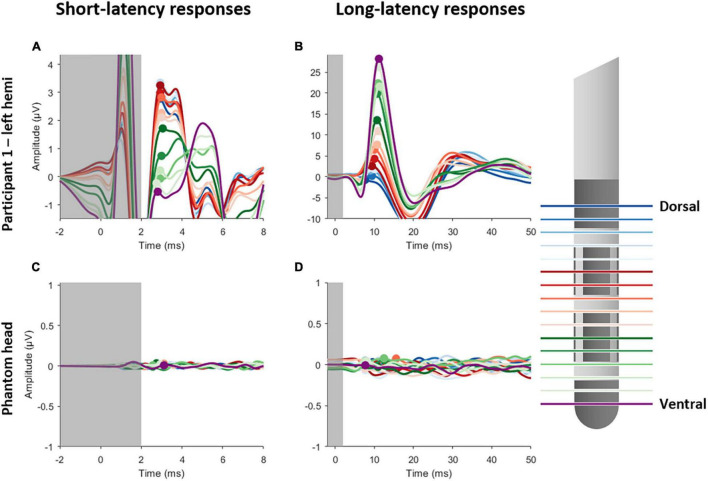
Short- and long-latency EPs recorded whilst employing MICC technology in steps of 20%, yielding a total of 16 EPs. Left panels show the short-latency EPs for participant 1 **(A)** and the phantom head **(C)**. Right panels show the long-latency EPs for participant 1 **(B)** and the phantom head **(D)**. Each EP is colored differently, as is indicated on the legend on the right side. The gray transparent box indicates the time window (–1 to 2 ms) where residual artifact might still be present. The peak amplitudes are indicated with a circle for P3 (left panels) and P10 (right panels).

Figure 1 shows the short- and long-latency EPs in response to DBS when using the MICC technology to vertically migrate the center of the electric field from the most dorsal DBS-contacts in 16 steps to the most ventral contact for a representative subject. Each of the 16 EPs are shown in a different color, as indicated in the legend. Figures 1A,B illustrate the short- and long-latency EPs recorded in participant 1 (left hemisphere), respectively, while Figures 1C,D the short- and long-latency EPs show recorded in a phantom head. All stimulation settings were well tolerated. In general, the EP morphology was similar to previously reported data recorded in a similar patient population (Walker et al., 2012; Miocinovic et al., 2018; Peeters et al., 2021). As expected, the P3 peak appeared strongest in the most dorsal DBS-contacts, while the P10 peak appeared strongest in the most ventral DBS-contacts (Peeters et al., 2021). Based on the analysis of the previous study (Peeters et al., 2021) we found a significant P3 peak in four out of five hemispheres and a significant P10 peak in all five hemispheres (see Supplementary Figures 1, 2). Therefore, further analysis on P3 was only performed in the four hemispheres where a significant P3 peak was detected. A summary of this analysis is provided in Supplementary Table 2.

### Distinct Evoked Potential Amplitudes Were Observed When Multiple Independent Current Control Was Used to Move the Center of the Electric Field to Location Between Two Vertically Adjacent Deep Brain Stimulation Contacts

**FIGURE 2:**
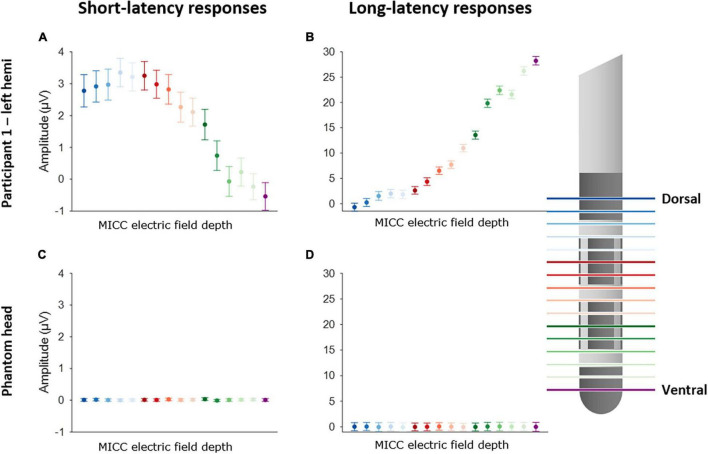
Effect of using MICC to change the center of the electric field on P3 and P10 amplitude. Left panels show the effect of MICC technology on P3 amplitude for participant 1 **(A)** and the phantom head **(C)**. Right panels show the effect on P10 amplitude for participant 1 **(B)** and the phantom head **(D)**. Each EP is colored differently, as is indicated on the legend on the right side. The dots show the mean peak amplitude (P3 or P10) calculated across all epochs (*n* = 400), the error bars show the 95% confidence interval (CI).

Figure 2 illustrates the change in EP amplitude for P3 peak (left panels) and P10 peak (right panels) for participant 1 (upper panels) and the phantom head (lower panels). Each of the 16 EPs are shown in a different color (see legend). A one-way ANOVA showed that there was a significant effect of the MICC-controlled electric field depth on P3 amplitude [*F*_(15, 6399)_ = 36.21; *p* < 0.0001] for participant 1. Additionally, a significant effect of MICC-controlled electric field depth on P10 amplitude [*F*_(15, 6399)_ = 395.57; *p* < 0.0001] was also found in this participant. Importantly, control stimulation in the phantom head showed no effect of MICC-controlled electric field depth on P3 nor P10 amplitude [P3: *F*_(15, 6399)_ = 0.30; *p* = 0.956; P10: *F*_(15, 6399)_ = 1.13; *p* = 0.3259] In total, we found that the MICC-controlled electric field depth had a significant effect on P3 amplitude in all four tested hemispheres and a significant effect on P10 amplitude in all five tested hemispheres (see Table 1 and Supplementary Figures 3, 4).

**TABLE 1:** Effect of MICC on P3 and P10 amplitude.

Participant no.	P3 (one-way ANOVA)		P10 (one-way ANOVA)	
	*P*-value	F-statistics	*P*-value	F-statistics
1L	<0.0001	36.21	<0.0001	395.57
1R	<0.0001	94.94	<0.0001	489.59
2L	<0.0001	3.71	<0.0001	18.31
3L	<0.0001	7.51	<0.0001	6.73
4L	–	–	<0.0001	229.87
Phantom head	NS	0.52	NS	0.33
Total (%)	4/4 (100%)		5/5 (100%)	

*L, left hemisphere tested; R, right hemisphere tested; NS, not significant; Total (%), total number of participants tested. One-way ANOVA was applied to evaluate if MICC technology significantly affected the P3 and P10 peak amplitudes as measured in each individual hemisphere.*

**FIGURE 3:**
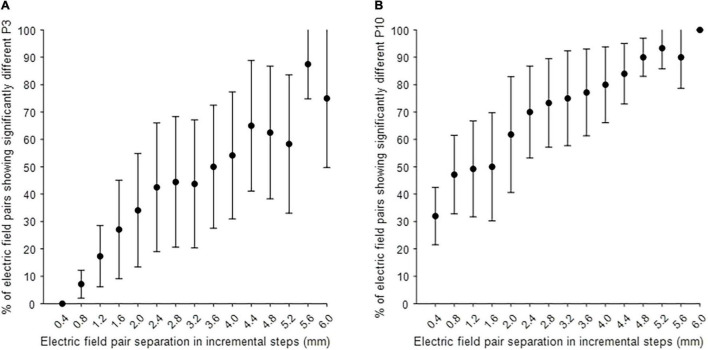
*Post hoc* analysis on the separability of electric field pairs on P3 and P10 amplitude. **(A)** The separability of MICC-controlled electric field depth on P3 amplitude in all tested hemispheres (*n* = 4). **(B)** The separability of MICC-controlled electric field depth on P10 amplitude in all tested hemispheres (*n* = 5). The x-axis shows the separation of electric field pairs in incremental steps (mm), varying from one step to fifteen steps with a proportional distance of 0.4 mm and the y-axis shows the percentage of electric field pairs showing a significantly different P3 or P10 peak amplitude (mean ± CI) in all tested hemispheres after Bonferroni correction was applied.

*Post hoc* analysis was performed to investigate the separability of MICC-controlled electric field depth on P3 and P10 amplitudes. Figure 3 shows the electric field pair separation in incremental steps, varying from one step to fifteen steps on the x-axis, and the percentage of electric field pairs showing a significantly different P3 (A) or P10 (B) peak amplitude (mean ± CI) on the y-axis for all tested hemispheres after Bonferroni correction was applied. The P10 peak was significantly different on around 30% of immediately adjacent electric field pairs (1 step separation) increasing to 100% of electric field pairs when the separation was increased to 15 steps (Figure 3B). The P3 peak was only significantly different on around 5% of electric field pairs when the separation was increased to 2 steps. This percentage of significantly different electric field pairs increased steadily to around 80% of pairs as the separation was increased to 15 steps.

### Correlation Between Evoked Potential Amplitudes and Image-Derived Lead and Contact Position

**FIGURE 4:**
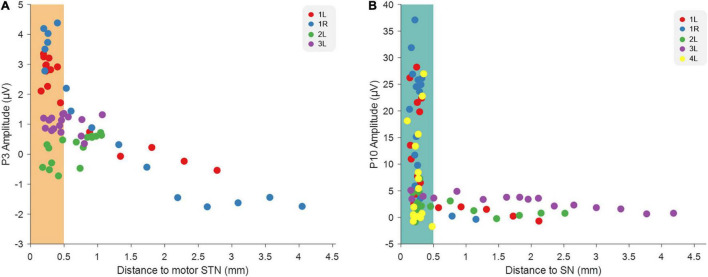
Relationship between EP amplitudes and the distance to image-derived anatomical structures. **(A)** The relationship between the distance of each MICC-controlled electric field depth to STN and the P3 amplitude recorded on that MICC-controlled depth in four hemispheres (*n* = 64). **(B)** The relationship between the distance of each MICC-controlled electric field depth to SN and the P10 amplitude recorded on that MICC-controlled depth in five hemispheres (*n* = 80). The colors indicate the sixteen MICC-controlled depths of the same hemisphere. When the distance was smaller than 0.5 mm, the electric field center was determined as “within” either dorsolateral STN (orange rectangle) or SN (blue rectangle).

The above results strengthen the idea published in a previous article (Peeters et al., 2021), stating that stimulation on the different depths preferentially modulates different nuclei, thereby causing the different EP peaks. We therefore plotted the average P3 and P10 amplitudes from all tested hemispheres as a function of the distance of each of the 16 electric field depths to dorsolateral STN and to SNr, respectively (Figure 4). This indicates indeed that the closer the MICC-controlled depth is to motor STN, the stronger the P3 peak amplitude appears and P10 peak amplitude appears strongest when stimulating from an MICC-controlled depth closest to SN.

## Discussion

We used MICC stimulation to vertically move the center of the electric field in fifteen incremental steps from the most dorsal DBS-contact to the most ventral DBS-contact while recording multichannel EEG EPs in PD patients. Thus, sixteen electric field locations were tested in total with a shift of approximately 0.4 mm per step calculated proportionally. In the four hemispheres were a P3 peak could be detected, incrementally changing the center of the electric field had a significant effect on the P3 amplitude. Furthermore, a P10 peak was detected in all five hemispheres and incrementally moving the electric field also had a significant effect on P10 amplitude. Importantly, in a control experiment using a phantom head, no P3 or P10 peaks were detected, nor was a significant effect of MICC on P3 or P10 peak amplitude detected when the stimulation location was moved. These results indicate that the small changes in vertical current steering can be achieved with MICC stimulation adjustments, and cause distinct neurophysiological responses. The center of the electric field in reference to the P3 and P10 peak amplitudes do not follow a straight line, which indicate that EP responses show a rather heterogeneous sensitivity to steering along the lead, an effect that is probably dependent on the lead positioning in the brain.

Previously, we have reported that P3 and P10 peak amplitudes were significantly different when stimulating on different directional contacts (vertical and horizontal current steering). Furthermore, we showed that stimulating on DBS-contacts closest to dorsolateral STN resulted in the largest P3 peak, while stimulating on DBS-contacts closest to SNr caused the largest P10 peak. Those results indicated that P3 may be a good predictor for the best DBS-contact to initiate programming in a new patient, while P10 might help predict which contacts will results in SNr-related side effects (Peeters et al., 2021). In the current study, we went one step further by investigating the more precise changes in programming possibilities that can be achieved with MICC stimulation, i.e., moving the center of the electric field to targets located between two adjacent DBS-contacts. Overall, our present results show that small incremental shifts in electric field location using MICC technology result in significant differences in P3 and P10 peak amplitudes. These distinct neurophysiological responses suggest that MICC technology can deliver measurably more precise stimulation in DBS patients. Group analysis furthermore indicated that the closer the center of the electric field is positioned to dorsolateral STN, the stronger P3 amplitude appears to be, while the closer the electric field center is positioned to SN, the stronger P10 amplitude appears.

*Post hoc* analyses showed that MICC technology can result in significantly distinct P3 peak amplitudes when comparing electric field pairs with just two steps (i.e., with a distance of only 0.8 mm) in between, and distinct P10 peak amplitudes when comparing two adjacent electric field pairs (i.e., with a distance of only 0.4 mm). Thus, results reported here indicate that MICC technology can significantly increase the resolution of vertical steering by at least 60% (0.4 mm compared to 1 mm dual-monopolar). A multicenter, randomized, controlled study has investigated MICC devices in a large population, where they found improvements in motor function and quality of life, while maintaining the safety profile in Parkinson’s disease patients. However, these clinicians were not able to assess the full spectrum of MICC on clinical outcomes (Vitek et al., 2020). Despite these promising results, it is therefore still not completely clear whether the more precise spatial targeting offered by MICC technology also results in improved therapeutic outcomes.

Similar to the previous study (Peeters et al., 2021), we found that P3 had the largest amplitude when stimulating from a MICC-controlled depth closest to dorsolateral STN, which suggest that P3 is associated with STN modulation. Furthermore, P10 had the largest amplitude when stimulating from a depth closest to SN, suggesting that P10 is associated with SN modulation (Figure 4). This strengthens the previous conclusion that different neural circuits are activated and that EPs thus might serve as a neurophysiological marker of STN-and SNr-DBS. On a clinical level, EPs could be used complementary to imaging approaches to guide DBS programming in individual patients.

One potential drawback of the increased parameter space offered by MICC technology is that is can be time consuming for the programmer to find the optimal center of the electric field. Imaging approaches can already offer a partial solution to this problem by suggesting hotspots where a programmer can begin. Our data now show that the P3 amplitude could offer a potential complimentary EEG-based approach. Furthermore, the study described here works further on previously reported study correlating P3 to dorsolateral STN (Peeters et al., 2021), provides more refined electrophysiological indication as to why we should direct the stimulation field toward dorsolateral STN.

There are some limitations to be noted for this study. We report here on data from just four patients (five hemispheres). However, even in this small group we found consistent results. All statistics on the effect of MICC on EP peak amplitude were also performed on an individual (hemisphere) level and it is important to note that DBS programming happens on a patient-specific level. Furthermore, the vertical steering was not performed in a randomized order due to time constraints. We believe that this method did not largely affect the results as low frequency DBS-EPs are similar regardless of time of capture in our dataset.

In conclusion, changing the electric field during electrical stimulation in STN in parkinsonian patients using MICC technology resulted in distinct EEG-based EP responses. More specifically, results indicate that MICC electric field pairs can produce statistically separable responses down to distances of approximately 0.8 mm or 0.4 mm. The results reported here enable future investigations to test whether these differences in electric field locations are also clinically distinct. Lastly, these results, together with those previously reported (Peeters et al., 2021), strengthen the idea that EPs may provide clinically relevant information to help guide programming in individual DBS patients.

## Data Availability Statement

The raw data supporting the conclusions of this article will be made available by the authors, without undue reservation.

## Ethics Statement

The studies involving human participants were reviewed and approved by KU Leuven/UZ Leuven Ethics committee. The patients/participants provided their written informed consent to participate in this study.

## Author Contributions

All authors listed have made a substantial, direct, and intellectual contribution to the work, and approved it for publication.

## Conflict of Interest

JP, AB, and TB were funded by Boston Scientific, VLAIO and EIT Health. BN received grants from Medtronic and Boston Scientific. The remaining authors declare that the research was conducted in the absence of any commercial or financial relationships that could be construed as a potential conflict of interest.

## Publisher’s Note

All claims expressed in this article are solely those of the authors and do not necessarily represent those of their affiliated organizations, or those of the publisher, the editors and the reviewers. Any product that may be evaluated in this article, or claim that may be made by its manufacturer, is not guaranteed or endorsed by the publisher.
