# Supplementary material for: Current Steering Using Multiple Independent Current Control Deep Brain Stimulation Technology Results in Distinct Neurophysiological Responses in Parkinson’s Disease Patients
Source: Front Hum Neurosci. 2022 Jun 2;16:896435. doi: 10.3389/fnhum.2022.896435 (PMC9203070; doi:10.3389/fnhum.2022.896435)
Supplement: Supplementary file 1 [file Data_Sheet_1.pdf]

Supplementary Material

Demographics and stimulation parameters

Patients that already participated in the previous study were now enrolled in a follow-up study, where we tested the MICC technology (see further). Four patients participated, one of which participated twice, yielding data from both hemispheres in this patient. In total, five hemispheres were tested. All participants were asked to refrain from taking their medication 12 hours prior to the study visit. Stimulation intensity was set at the top of therapeutic window (i.e. the highest stimulation intensity that did not evoke non-transient side effects).The pulse width and the stimulation frequency were kept constant at 60 µs and 10 Hz, respectively. Demographic data and stimulation parameters used during the experiment are summarized in Supplementary table 1.

**Participant No.**	**Gender**	**Age (yrs)**	**Stimulation intensity (mA)**
1L	F	50	4.0
1R	F	50	6.0
2L	M	55	5.0
3L	M	71	4.0
4L	M	47	6.0
Average	1F/3M	55.8	5.0

Legend*: L: left hemisphere tested; R: right hemisphere tested*

Supplementary table 1: Demographic data & Stimulation parameters

Short- and long-latency EP in response to stimulation at different depths using MICC technology

Supplementary figures 1 and 2 illustrate the short- and long-latency EPs in response to DBS when using the MICC technology to incrementally change the center of the electric field from the most dorsal DBS-contacts in 15 steps to the most ventral contacts in all tested hemispheres (participant 1 left hemisphere shown in research article). The short-latency EPs were recorded via the motor cortex EEG channel ipsilateral to stimulation (i.e., EEG channel F3 for the left hemisphere and F4 for the right hemisphere). The long-latency EPs were recorded via the prefrontal cortex EEG channel ipsilateral to stimulation (i.e., EEG channel AF7 for the left hemisphere and AF8 for the right hemisphere). Based on the analysis of the previous study (Peeters et al. 2021) we found a significant P3 peak in four hemispheres and a significant P10 peak in all five hemispheres. Supplementary table 2 shows a recap of this analysis.

**Participant No.**	**Participant No. in previous study**	**Intensity P-value (F-statistics)**	
		**P3**	**P10**
1L	1L	< 0.0001 (22.2)	< 0.0001 (862.2)
1R	1R	< 0.0001 (257.8)	< 0.0001 (770.9)
2L	2L	NS (1.48)	< 0.0001 (34.5)
3L	5L	0.0002 (8.6)	< 0.0001 (11.8)
4L	6L	NS (0.12)	< 0.0001 (267.1)
Total (%)	-	4/6 (67%)	6/6 (100%

Supplementary table 2: Recap of ANOVA results investigating the effect of stimulation intensity on P3 and P10 peak amplitude

Distinct EP amplitudes were observed when MICC was used to move the center of the electric field center to location between two vertically adjacent DBS-contacts

Supplementary figure 3 illustrates the change in EP amplitude for the P3 peak amplitudes. Each of the 16 EPs are shown in a different color (see legend). ANOVA showed a significant effect of MICC controlled depth on P3 amplitude ($F_{(15,6399)} = 94.94$; $p < 0.0001$) for the right hemisphere of participant 1, ($F_{(15,6399)} = 3.71$; $p < 0.0001$) for the left hemisphere of participant 2 and ($F_{(15,6399)} = 7.51$; $p < 0.0001$) for the left hemisphere of participant 3. Thus, MICC significantly affected P3 amplitude in all five tested hemispheres (left hemisphere of participant 1 shown in research article).

Supplementary figure 4 shows the change in EP amplitude for the P10 peak amplitudes. Each of the 16 EPs are shown in a different color (see legend). ANOVA showed a significant effect of MICC controlled depth on P3 amplitude ($F_{(15,6399)} = 489.59$; $p < 0.0001$) for the right hemisphere of participant 1, ($F_{(15,6399)} = 18.31$; $p < 0.0001$) for the left hemisphere of participant 2, ($F_{(15,6399)} = 12.86$; $p < 0.0001$) for the right hemisphere of participant 2, ($F_{(15,6399)} = 5.14$; $p < 0.0001$) for the left hemisphere of participant 3 and ($F_{(15,6399)} = 229.87$; $p < 0.0001$) for the left hemisphere of participant 4. Thus, MICC significantly affected P10 amplitude in all five tested hemispheres (lef themisphere of participant 1 shown in research article).

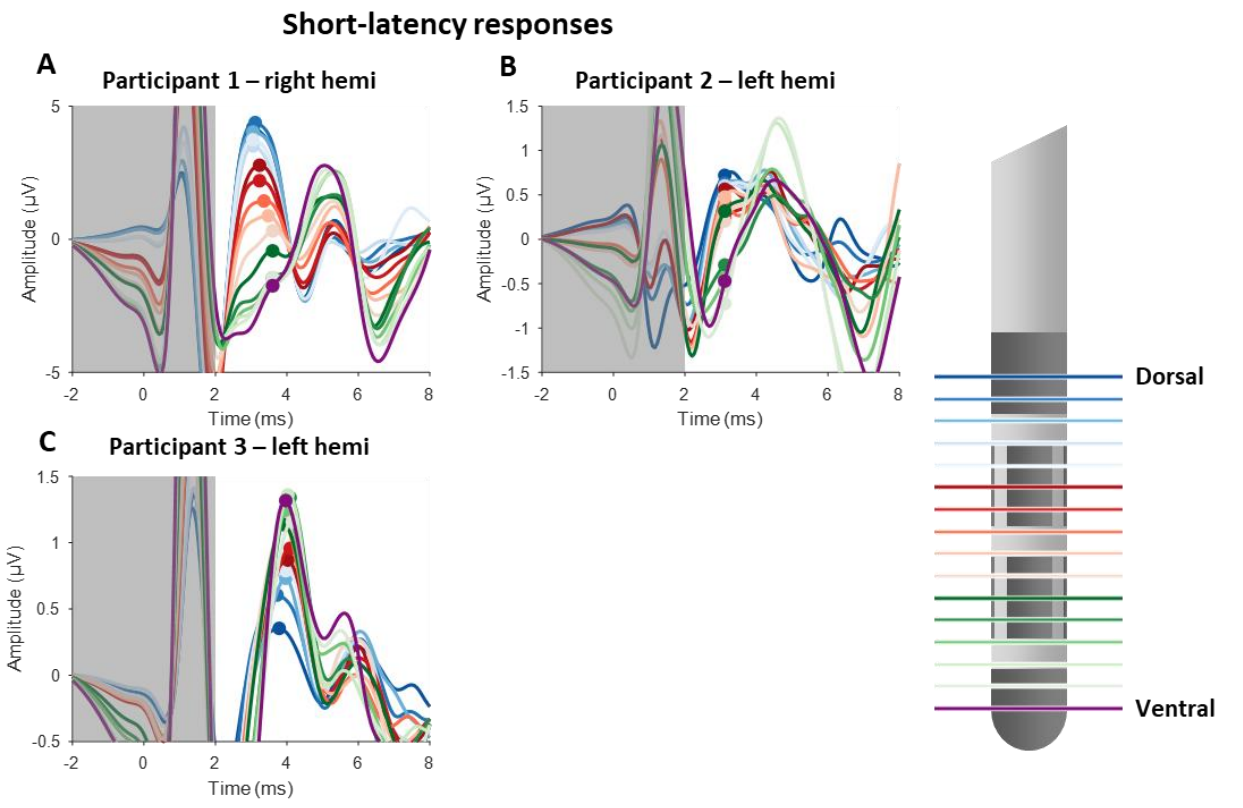

Supplementary figure 1: Short-latency EPs recorded whilst employing MICC technology in 16 incremental steps in the right hemisphere of participant 1 (A), the left hemisphere of participant 2 (B) and the left hemisphere of participant 3 (C). Each EP is colored differently, as is indicated on the legend on the right side. The grey transparent box indicate the time window (-1 to 2 ms) where residual artifact might still be present. The peak amplitudes are indicated with a circle.

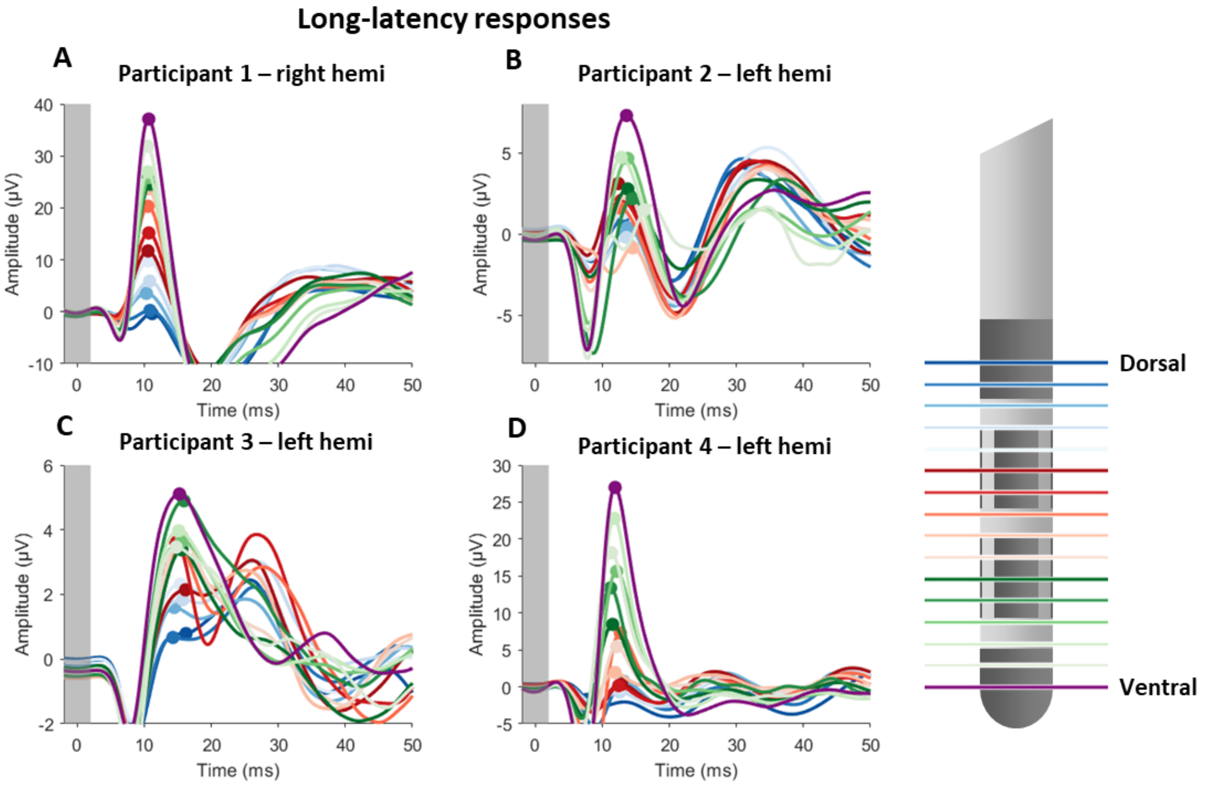

Supplementary figure 2: Long-latency EPs recorded whilst employing MICC technology in 16 incremental steps in the right hemisphere of participant 1 (A), the left hemisphere of participant 2 (B), the left hemisphere of participant 3 (C) and the left hemisphere of participant 4 (D). Each EP is colored differently, as is indicated on the legend on the right side. The grey transparent box indicate the time window (-1 to 2 ms) where residual artifact might still be present. The peak amplitudes are indicated with a circle.

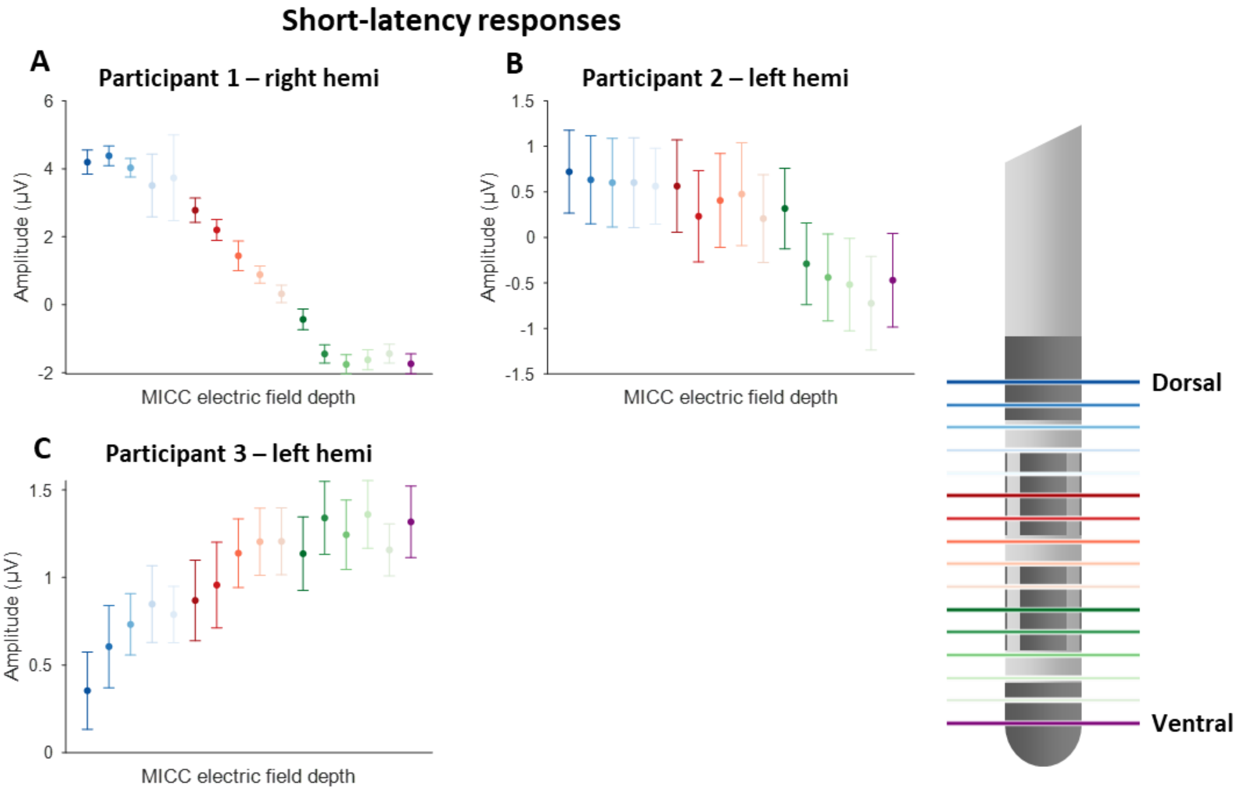

Supplementary figure 3: Effect of MICC technology on P3 amplitude for the right hemisphere of participant 1 (A), the left hemisphere of participant 2 (B) and the left hemisphere of participant 3 (C). Each EP is colored differently, as is indicated on the legend on the right side. The dots show the mean peak amplitude (P3 or P10) calculated across all epochs (n = 400), the error bars show the 95% confidence interval (CI).

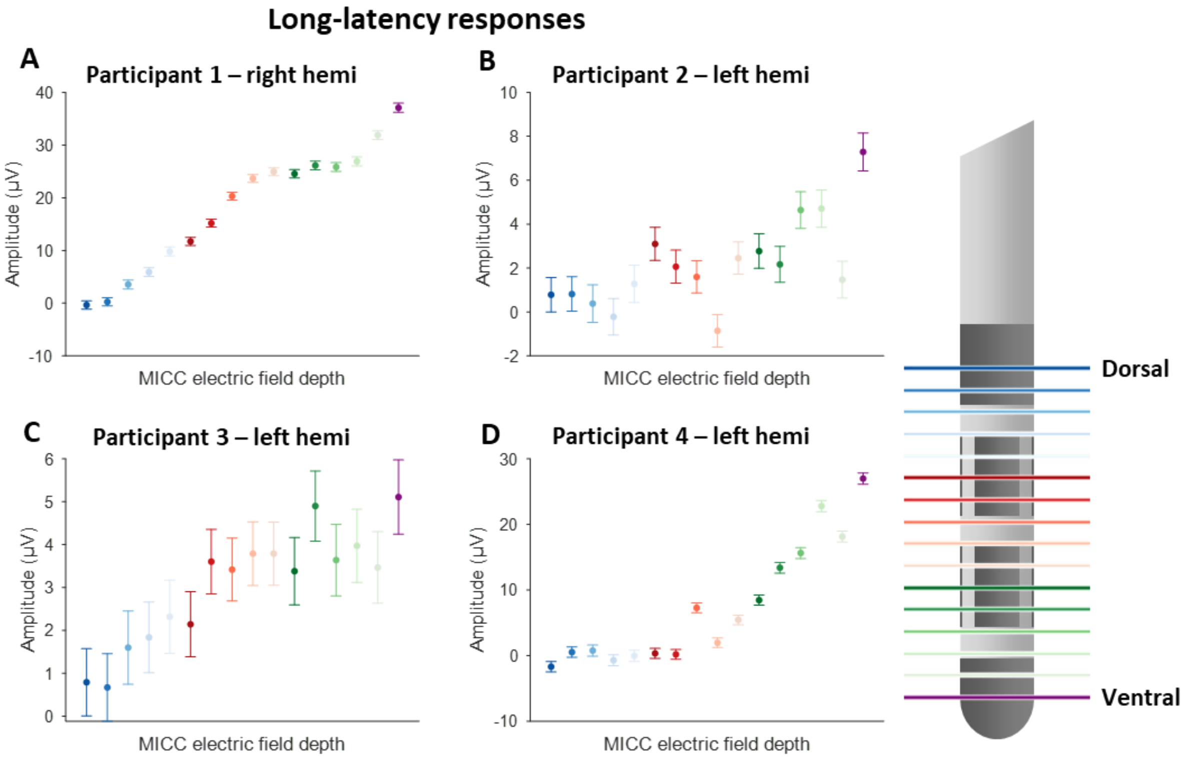

Supplementary figure 4: Effect of MICC technology on P10 amplitude for the right hemisphere of participant 1 (A), the left hemisphere of participant 2 (B), the left hemisphere of participant 3 (C) and the left hemisphere of participant 4 (D). Each EP is colored differently, as is indicated on the legend on the right side. The dots show the mean peak amplitude (P3 or P10) calculated across all epochs (n = 400), the error bars show the 95% confidence interval (CI).

Correlation between EP amplitudes and image-derived electric field center position

Supplementary figure 5 illustrates the correlation between the EP amplitudes recorded when stimulating from the 16 different electric field centers to relevant anatomical regions of the left (A) and right (B) hemisphere of participant 1 and the left hemispheres of participant 2 (C), participant 3 (D) and participant 4 (E). The left panels show the lead position visualized using Lead-DBS where STN and SN are visualized. The right top panels show the correlation between P3 amplitude and the distance to motor STN and the right bottom panels show the distance between P10 amplitude and the distance to SN. Each of the 16 EPs are shown in a different color (see legend). In general, we can observe that P3 appears strongest when stimulating closer to motor STN and that P10 appears strongest when stimulating closer to SN, similar to what has been published in the previous article by Peeters et al. 2022.

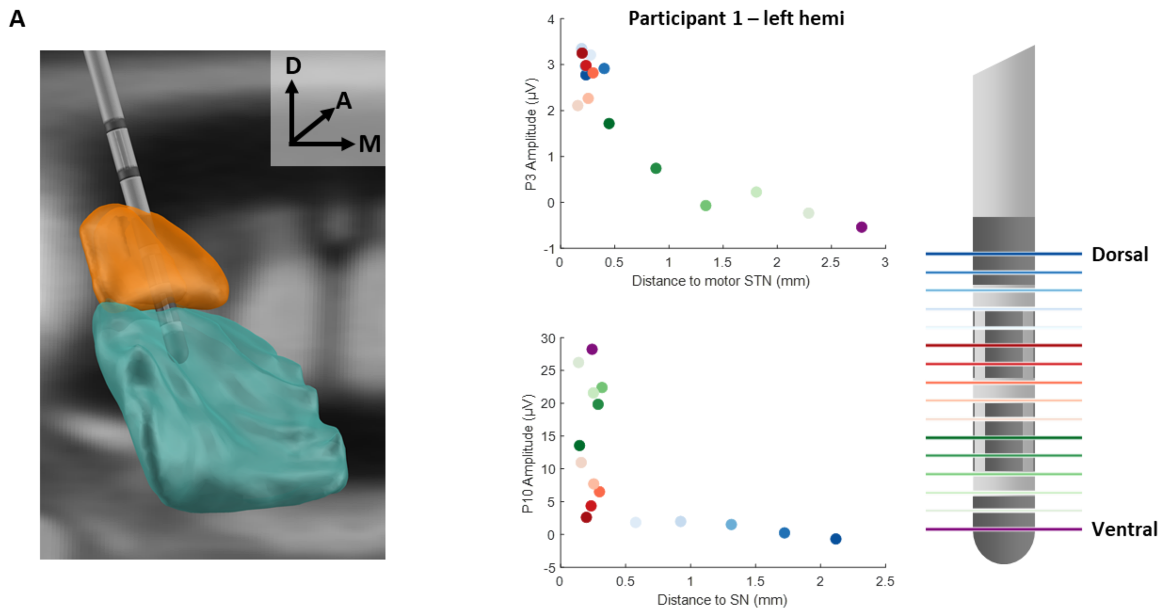

B

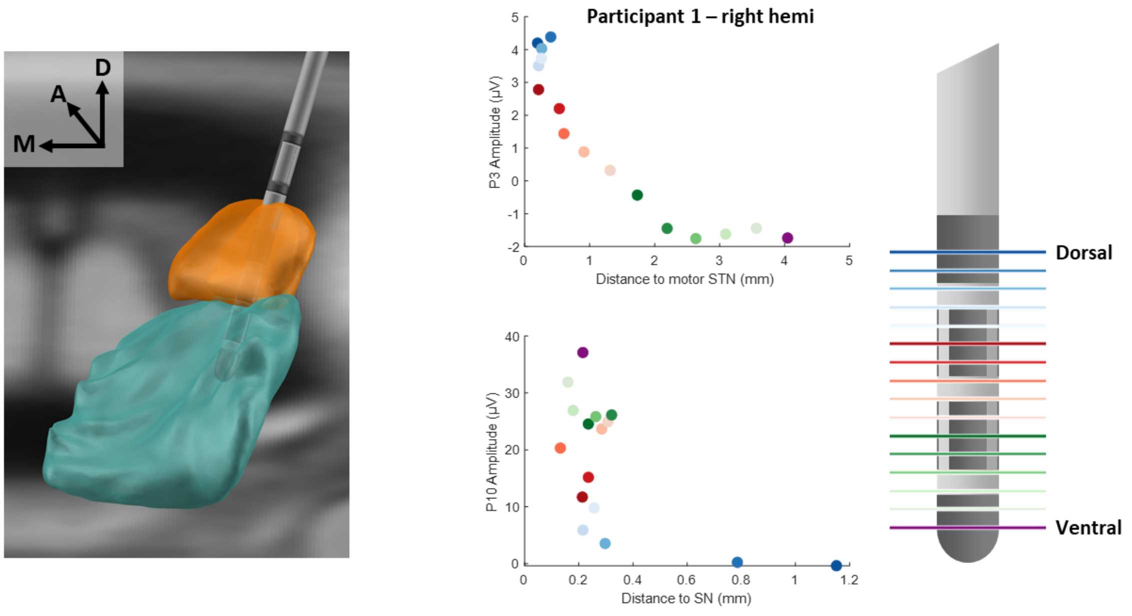
D
A
M
Participant 1 – right hemi
P3 Amplitude (µV)
Distance to motor STN (mm)
P10 Amplitude (µV)
Distance to SN (mm)
Dorsal
Ventral

C

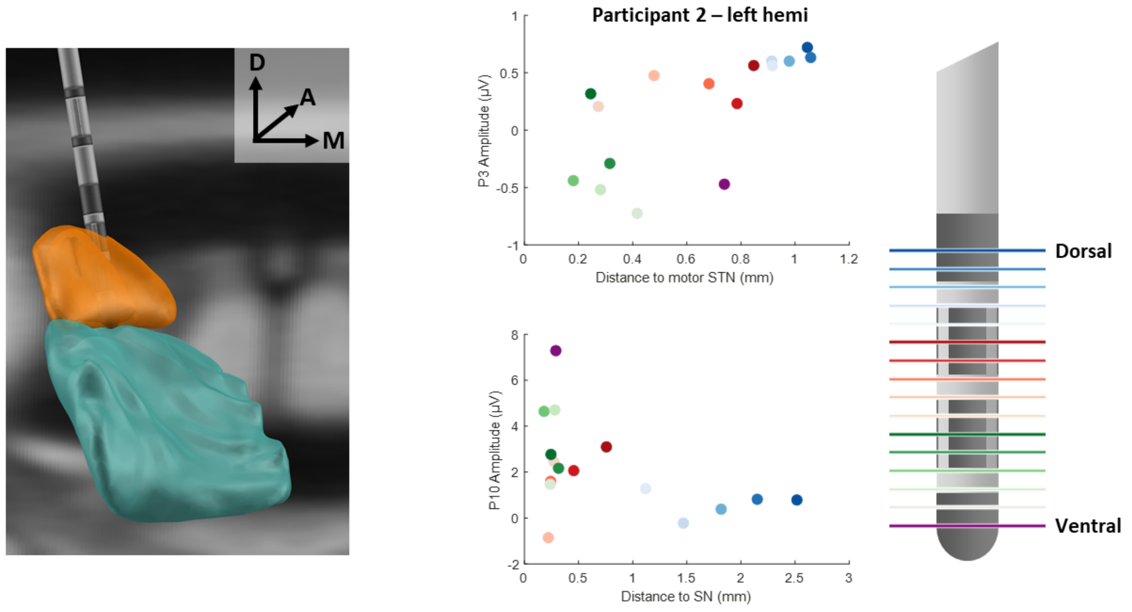
D
A
M
Participant 2 – left hemi
P3 Amplitude (µV)
Distance to motor STN (mm)
P10 Amplitude (µV)
Distance to SN (mm)
Dorsal
Ventral

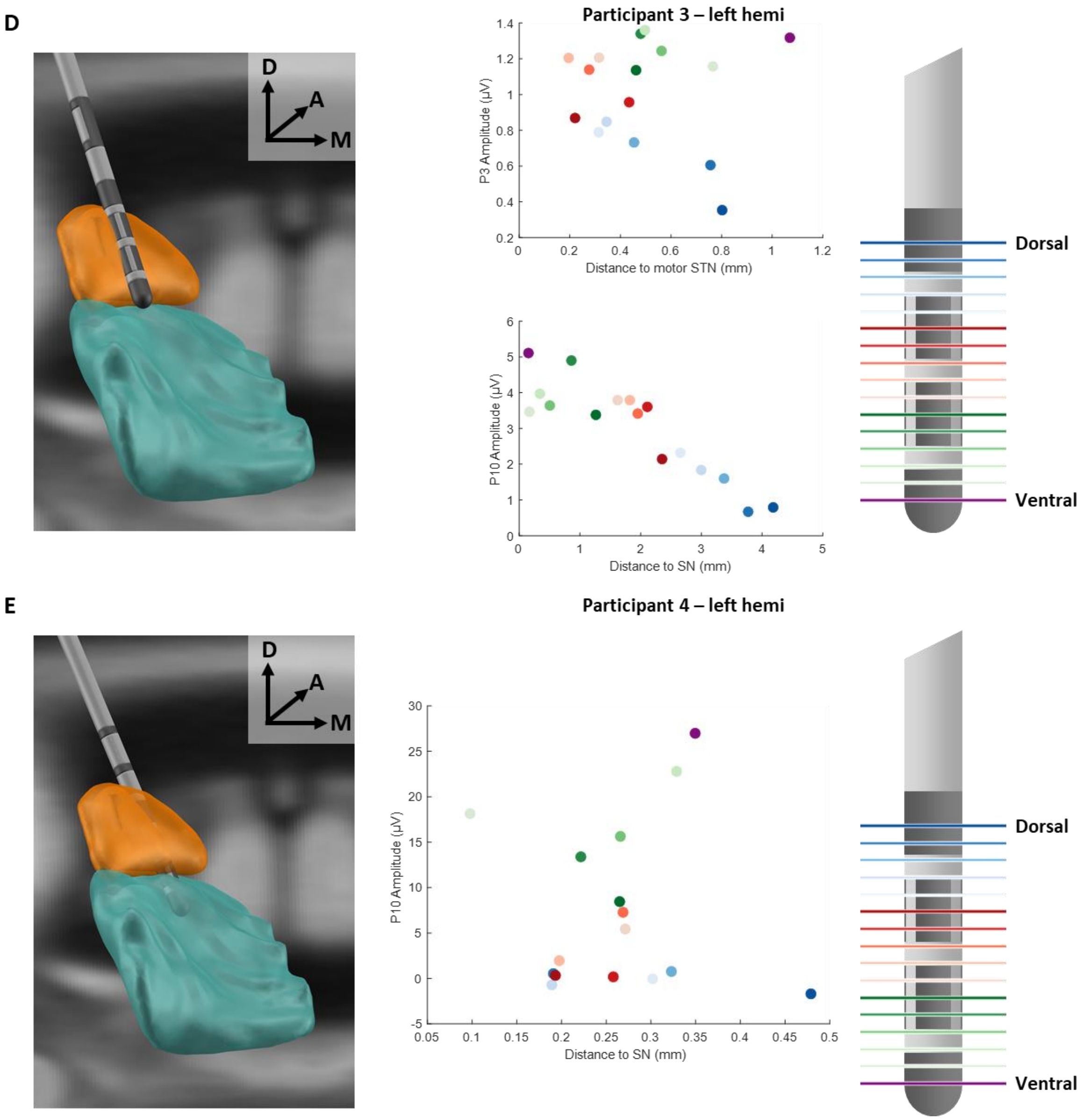

Supplementary figure 5: Relationship between EP amplitudes and image-derived electric field center position for the left (A) and right (B) hemisphere of participant 1, and the left hemispheres of participant 2 (C), participant 3 (D) and participant 4 (E). Left panel: lead positioning in reference to the relevant anatomical regions (STN: orange; SN: green). Top right panel: correlation between the distance from each electric field center to motor STN (in mm) to the P3 peak amplitude (in µV) recorded when stimulating from each electric field center. Bottom right panel: correlation between the distance from each electric field center to SN (in mm) to the P10 peak amplitude (in µV) recorded when stimulating from each electric field center (bottom). Each EP is colored differently, as is indicated on the legend on the right side. The dots show the mean peak amplitude (P3 or P10) calculated across all epochs (n = 400). (legend: D: dorsal, A: anterior, M: medial).
